# Post-synaptic Release of the Neuronal Tissue-Type Plasminogen Activator (tPA)

**DOI:** 10.3389/fncel.2019.00164

**Published:** 2019-04-24

**Authors:** Sophie Lenoir, Alexandre Varangot, Laurent Lebouvier, Thierry Galli, Yannick Hommet, Denis Vivien

**Affiliations:** ^1^Physiopathology and Imaging of Neurological Disorders, UNICAEN, INSERM, UMR-S U1237, Normandie Université, Caen, France; ^2^Membrane Traffic in Healthy & Diseased Brain, Center of Psychiatry and Neurosciences, INSERM U894, Sorbonne Paris-Cité, Université Paris Descartes, Paris, France; ^3^Department of Clinical Research, Caen University Hospital, CHU Caen, Caen, France

**Keywords:** tissue-type plasminogen activator, trafficking, vesicles, synapses, neurons

## Abstract

The neuronal serine protease tissue-type Plasminogen Activator (tPA) is an important player of the neuronal survival and of the synaptic plasticity. Thus, a better understanding the mechanisms regulating the neuronal trafficking of tPA is required to further understand how tPA can influence brain functions. Using confocal imaging including living cells and high-resolution cell imaging combined with an innovating labeling of tPA, we demonstrate that the neuronal tPA is contained in endosomal vesicles positives for Rabs and in exosomal vesicles positives for synaptobrevin-2 (VAMP2) in dendrites and axons. tPA-containing vesicles differ in their dynamics with the dendritic tPA containing-vesicles less mobile than the axonal tPA-containing vesicles, these laters displaying mainly a retrograde trafficking. Interestingly spontaneous exocytosis of tPA containing-vesicles occurs largely in dendrites.

## Introduction

Tissue-type plasminogen activator (tPA) is a serine protease which was first shown to be produced by endothelial cells and released in the blood stream, where it plays roles in clotting and fibrinolysis (Astrup and Stage, 1952). Most of its vascular functions are associated with its ability to promote the conversion of plasminogen, bound to fibrin or cells, into plasmin (Hoylaerts et al., 1982; Briens et al., 2017). In the brain parenchyma, tPA is expressed in neurons and possibly in a subset of glial cells (Tsirka et al., 1995; Hultman et al., 2008; Louessard et al., 2016). It is involved in cell migration, neuronal plasticity, neuronal survival, maintenance of the blood-brain barrier integrity and in the control of inflammatory processes (Hébert et al., 2016). When released into the extracellular space, tPA mediates generation of plasmin which in turn regulates the degradation of the extracellular matrix (Baricos et al., 1995) and the conversion of growth factors, chemokines precursors, neurotrophins into their active forms (Bruno and Cuello, 2006; Lee et al., 2007; Su et al., 2008; Rodier et al., 2014). For example, tPA was reported to promote the conversion of pro-BDNF (Brain-Derived Neurotrophic Factor) into its mature form (m-BDNF) through a plasmin-dependent mechanism (Pang et al., 2004). tPA can also bind to various receptors including the N-methyl-D- aspartate receptor (NMDAR), the low-density lipoprotein receptor-related protein (LRP), Platelet Derived Growth Factor-CC and annexin-II (Hajjar et al., 1994; Nicole et al., 2001; An et al., 2008; Su et al., 2008). tPA is thus now considered as a neuromodulator and the mechanisms that govern its distribution at the synapse should play an important role. As such, we showed in a previous study (Fernández-Monreal et al., 2004) that the tPA released by neurons upon depolarization was recaptured by astrocytes, through a LRP-dependent mechanism (Cassé et al., 2012). In turn, astrocytes can release tPA at the synaptic cleft by a mechanism dependent on the concentration of extracellular glutamate (Cassé et al., 2012). More recently, we revealed that, when released from neurons, the tPA can promote the conversion of pro-BDNF into m-BNDF by a mechanism dependent on the activation of plasminogen bound to the surface of astrocytes (Briens et al., 2017).

Although tPA was reported to be present at the synapse (Wu et al., 2015), co-immunoisolated with synaptobrevin-2 (VAMP2) positive vesicles (Louessard et al., 2016) and associated with an increased synaptic endocytosis (Yepes et al., 2016), its localization at the synapse and the mechanisms that govern its neuronal trafficking are largely unknown. The present study attempted to examine in detail the neuronal trafficking of tPA (Lenoir, 2018). The results indicate that: (i) the tPA is present in VAMP2 positives vesicles in axons and dendrites; (ii) with the pre-synaptic dendritic vesicles that contain tPA that are more statics than the axonal vesicles that are also positives for tPA; (iii) in basal conditions the release of tPA-containing vesicles is higher in dendrites than in axons and; (iv) the tPA is also present in endosomal vesicles that are positives for Rabs.

## Materials and Methods

### Reagents

Recombinant human tPA (Actilyse®) was purchased from Boehringer Ingelheim (Ingelheim am Rhein, Germany). Fetal bovine serum, horse serum, lipofectamine® 2000 reagent, B27 supplement, glutamine, laminin, neurobasal medium, and penicillin/streptomycin were purchased from ThermoFisher (Waltham, Massachusetts, USA). Dulbecco's modified Eagle's medium (DMEM), poly-D-lysine, phosphate-buffered saline (PBS), paraformaldehyde, albumin from bovine serum, ammonium chloride (NH_4_Cl), potassium chloride (KCl), and rabbit polyclonal antibody were purchased from Sigma-Aldrich (St Louis, MO, USA). Aprotinin (Trasylol) was purchased from Bayer (Leverkusen, Germany). HaloTag® TMR ligand was purchased from Promega (Madison, Wisconsin, USA). Human Embryonic Kidney HEK-293 cells were purchased from ATCC (Teddinghton, UK). The following primary antibodies were used for immunocytochemistry: mouse monoclonal anti-HaloTag® (dilution 1:1,000; Promega; G9211) rabbit anti-tPA polyclonal antibody (dilution 1:1 500; generous gift from R. Lijnen, Leuven), chicken anti-Microtubule-associated protein 2 (MAP2) polyclonal antibody (dilution 1:8 000; Abcam, Cambridge, UK; ab5392), rabbit anti-tau polyclonal antibody (dilution 1:500; Abcam; ab64193), guinea pig anti-synapsin polyclonal antibody (dilution 1:500; Synaptic System; 106 004) and rabbit anti-homer-1C polyclonal antibody (dilution 1:500; Synaptic System; 160 003). Primary antibodies were used for immunoblotting are: anti-tPA (dilution 1:10 000; rabbit polyclonal antibody; Abcam; ab62763) and anti-HaloTag® (dilution 1:1,000; mouse monoclonal antibody; Promega; G9211). Secondary fluorescent antibodies (Alexa647 or Cy2; dilution 1:800) were purchased from Jackson Immunoresearch (Bar Harbor, ME, USA) and secondary peroxidase antibodies for immunoblotting: anti-mouse (IgG A9044 1:80,000), anti-rabbit (IgG A0545 1:80,000) from Sigma-Aldrich (St Louis, MO, USA).

### Plasmid Constructs

The cDNA encoding for amino acids 1 to 32 of the peptide signal of human tPA was amplified from the full-length human tPA cDNA. The corresponding Polymerase Chain Reaction (PCR) product was subcloned into the eukaryotic expression plasmid pCDNA5.1 between NheI and BamHI. Then, the full length coding sequence for mature human tPA was PCR amplified and subcloned downstream of the “peptide signal−6xHis” between BamHI and XhoI restriction sites to generate a cDNA encoding for the mature 6xHis human tPA (tPAwt). The cDNA of HaloTag® without the Tobacco Etch Virus protease (TEV) cleavage site was PCR amplified from the plasmid pFC14A (Promega, France) and subcloned into pCDNA5.1-tPA between the end of the sequence encoding for tPA and the STOP codon at the end of the Halo-Tag®, then fused in BamHI between the His-tag and the beginning of the cDNA for tPA (HD-Fusion technologies from Clontech, USA). For synapsin promote-driven constructs, we replaced the CMV promotor of the plasmid pCI by the cDNA of the synapsin promotor subcloned between BglII and NheI. For pCMV_HaloTag®-tPA-SEP, the cDNA encoding for the pH sensitive GFP (pHluorin) was subcloned by fusion into pCMV_HaloTag®-tPA between the end of the sequence of tPA and the STOP codon. The constructs pCMV_GFP-homer-1C, pCMV_GFP-VAMP2, pCMV_GFP-Rab pCMV_CFP-Rab and pCAG_RFP were provided by Thierry Galli (Paris, France). pCMV_GFP (pAcGFP1-N1 Vector, Cat#632469) and pPLAT_GFP were purchased from Takara Bio (Kusatsu, Japan) and Genecopoeia (Rockville, Maryland, USA), respectively. All the constructs were amplified in *Escherichia coli* DH5α cells and purified by a Nucleobond endotoxin-free plasmid DNA PC 2000 kit (Macherey-Nagel) according to the manufacturer's instructions.

### Immunobloted

Fifty nanograms of tPA (Actilyse®), tPA-HaloTag® (coming from tPA-HaloTag®-transfected HEK cells) were loaded in 10% polyacrylamide gel. Polyacrylamide gels were then transferred on a PVDF membrane and immunoblotted with the primary antibodies. After incubation with the secondary antibodies, proteins were visualized with an enhanced chemiluminescence western blot detection reagent (ECL Prime Western Blotting System; GE Healthcare; RPN2232) using ImageQuant LAS 4000 Camera (GE Healthcare, Chicago, IL, USA).

### Fibrin-Agar Zymography Assay

The presence of active tPA-HaloTag® (coming from tPA-HaloTag®-transfected HEK-293 cells) was detected by laying the sodium dodecyl sulfate polyacrylamide gel onto a fibrin-agar layer containing plasminogen (SDS-PAGE) performed as previously described (Gaussem et al., 1993). Fifty nanograms of purified tPA-HaloTag® and Actilyse® (control) were subjected to SDS electrophoresis (10% polyacrylamide gels, under non-reducing conditions). SDS was then exchanged with 2.5% Triton X-100. After washing off excess Triton X-100 with distilled water, the gel was carefully overlaid on a 1% agarose gel containing 1 mg/mL bovine fibrinogen, 100 nM plasminogen and 0.2 NIH U/mL of bovine thrombin. Zymograms were allowed to develop at 37°C for 12 h and photographed at regular intervals using dark-ground illumination.

### Cell Cultures

Cortical astrocyte cell cultures were prepared from 1 to 3 days postnatal mice. Cerebral cortices were dissected and dissociated in DMEM. Then, cells were plated in DMEM supplemented with 10% fetal bovine serum, 10% horse serum and 2 mM glutamine on poly-D-lysine (0.1 mg/ml) and laminin (0.02 mg/ml)-coated T75 Flasks. The medium was changed two times weekly until the cell reach confluence, after cells were maintained in DMEM supplemented with 5% fetal bovine serum, 5% horse serum and 2 mM glutamine. To maintain neuronal cells without serum (glio-conditioned medium), astrocytes cultures are incubated over night with Neurobasal Medium supplemented with 0.4 mM glutamine, 2% B27 supplement 50X and penicillin streptomycin (10,000 IU/ml; 10,000 UG/ml).

Primary cultures of cortical or hippocampal neurons were prepared from fetal mice (embryonic day 14) as previously described (Buisson et al., 1998). Cortices or hippocampi were dissected and dissociated in DMEM and plated (250 000 cell/ mL) on glass bottom microwell dishes (MatTek Corporation, P35G-1.5-14-C, Ashland, MA, USA) earlier coated with poly-D-lysine (0.1 mg/ml) and laminin (0.02 mg/ml). Cells were cultured in Neurobasal Medium supplemented with 0.4 mM of glutamine, 2% B27 supplement 50X, 10% horse serum and penicillin/streptomycin (10,000 IU/ml; 10,000 UG/ml). After 1 h, media were replaced by glio-conditioned medium obtained from primary cultures of astrocytes (see above). Cultures were maintained at 37 °C in a humidified 5% CO_2_ atmosphere. One third of medium was changed one time weekly by fresh glio-conditioned medium.

### Neuronal Transfection

Transfections were performed at Day *In Vitro* (DIV) 12 or DIV 20. Neuronal cultures were washed with HEPES and Bicarbonate Buffered Salt Solution (HBBSS; NaCl: 116 mM, KCl: 5.4 mM, CaCl_2_: 1.8 mM, MgSO_4_: 0.8 mM, HEPES: 12 mM, NaH_2_PO_4_: 0.34 mM, D-glucose: 5.5 mM, NaHCO_3_: 25 mM and Glycine: 10 μM) prior a 8 h incubation in the presence of the mentioned cDNAs and lipofectamine® 2000-containing HBSS, HBSS is then replaced by regular media as described above (cell cultures section). The transfection efficiency is 10–20%.

### tPA-HaloTag® Detection With HaloTag® TMR Ligand

After 24–48 h, neuronal cultures were washed with HBBSS, HaloTag® TMR ligand was added during 15 min and neuronal cultures were washed again with HBBSS (to remove unbound ligand).

### Immunocytochemistry

Neuronal cultures were washed with HBBSS, fixed in paraformaldehyde 4% for 20 min at room temperature, washed in PBS (0.1 M) and blocked 1 h in PBS containing 0.3% Triton X100 and albumin (4%). The primary and secondary fluorescent antibodies were used (see reagent section) and confocal laser-scanning microscopy was performed (see the next section).

### Laser Scanning Confocal Microscopy

Laser-scanning confocal microscopy (LSCM) was performed using an inverted Leica SP5 confocal microscope (Leica Microsystems SAS; Leica, Wetzlar, Germany) equipped with an Argon Gas laser and a X40 NA = 1.3 oil immersion objective. Culture was scanned at room temperature with 458-, 488-, 561, 633-nm, laser lines to detect the CFP, GFP or SEP or Cy2, HaloTag® TMR ligand and Alexa647, respectively and high-resolution images (1024 × 1024, 12 bits) of optical sections (z stack, step: 0.45 μm) were captured using sequential line (mean of three) scanning. Colocalizations of two or three fluorophores were qualitatively assessed in the x, y, and z planes of each optical section. Maximal projection images of confocal z series (stacks) were generated where indicated in the figure legends. Minimal adjustments to image contrast and intensity were made in ImageJ software using the levels or contrast/brightness functions.

### Live Imaging

Time-lapse images were collected at a 512 × 512–pixel resolution (8 bit). A short interval (0.648 s) for a total of 465 images in a non-sequential mode was used to maximize vesicles resolution trajectories. A relatively low laser intensity was used to minimize laser-induced cellular damage. All acquisitions were performed using a temperature control system (“cube & box,” life imaging services, Basel, Switzerland) at 37°C.

### STED Microscopy

Confocal and STED images were acquired with a Leica TCS SP8 Confocal/STED 3 × microscope with an oil-immersion 100 ×, 1.44-N.A. objective. Dual-color sequential confocal scans were followed by dual-color sequential STED scans by using a STED microscope. HaloTag® TMR ligand and GFP were excited with 555 and 488-nm white light lasers respectively, at 2–5% of laser power in this particular order. During STED scanning, GFP, and HaloTag® TMR ligand signals were depleted with 592 nm and 660 nm time-gated depletion lasers.

### Image Analysis

Confocal images were analyzed with the ImageJ software. The numbers of vesicles per 100 μm (**Figures 3, 5, 6** and Supplementary 4) were analyzed with the “cell counter” (for the number of puncta) and “straight line” functions (for the distance). Localization of tPA-HaloTag® at the synapse was determined with the “RGB plot profile” function (**Figure 4**). Colocalization between tPA-HaloTag® and other proteins was studied with the “Pearson's correlation” function (Plugins JACoP; **Figures 5, 7**) and the “Cell counter” function was used to calculate the percentage of colocalization (**Figures 5, 7**). Live –cell video-acquisitions of tPA-HaloTag® and Halotag®-tPA-SEP were analyzed using kymographs generated using kymographbuilder plugin for ImageJ with a variable length between of 60 to 100 μm (x-axis) and a total time of 5 min (y-axis) to extract kinetics parameters: velocity (μm/s), dynamic (**Figure 3**, **6** and Supplementary 3, 4). For the flow analysis, all kymographs were generated with a length of 60 μm and a total time of 5 min. Imaris x64 7.7.2 software (Bitplane) was used to model the two configurations of tPA at the synapse: pre-synaptic tPA and post-synaptic tPA (**Figure 4**).

### Analysis of tPA Containing Vesicle Motility

tPA-HaloTag® motility analysis was analyzed in both axonal and dendritic processes. Axons were distinguished from dendrites firstly based on known morphological characteristics: greater length, thin and uniform diameter, and sparse branching (Banker and Cowan, 1979). After a dozen retrospective immunostainings with an anti-MAP2 antibody of each neurite, a kymograph type profile for each neuronal process has been established and used as a basis for neurite distinction. We selected the proximal region of axons and dendrites for time-lapse imaging analysis. Only those that appeared to be single axons and separate from other processes in the field were chosen for recording axonal tPA-HaloTag® transport. Regions where crossing or fasciculation occurred were excluded from analysis. A tPA-HaloTag® vesicle has been considered stationary if it remained immotile for the entire recording period; a vesicle has been considered mobile only if the displacement was ≥2 μm during 5 min.

### Experimental Design and Statistical Analysis

The values of charts are presented as mean ± SEM. All analyses were completed from a minimum of two to nine independent cultures. Each experiment was performed from independent dish of cultured cortical neurons. The number of sample, named “n” corresponds to either the number of independent neurons analyzed (all figures excepted **Figure 3**) or to the number of independent vesicles analyzed (**Figure 3**). All statistical analyses were performed with the Statistica® software. A non-parametric Mann-Whitney U test was performed for all analyses unless for charts D and G of the **Figure 3** for which we used a Student's *t*-test. Vesicular intensities in NH_4_Cl experiments were analyzed with a non-parametric Wilcoxon signed-rank test for matched samples (Supplementary 3).

## Results

### Axonal and Dendritic Trafficking of tPA

**Figure 1:**
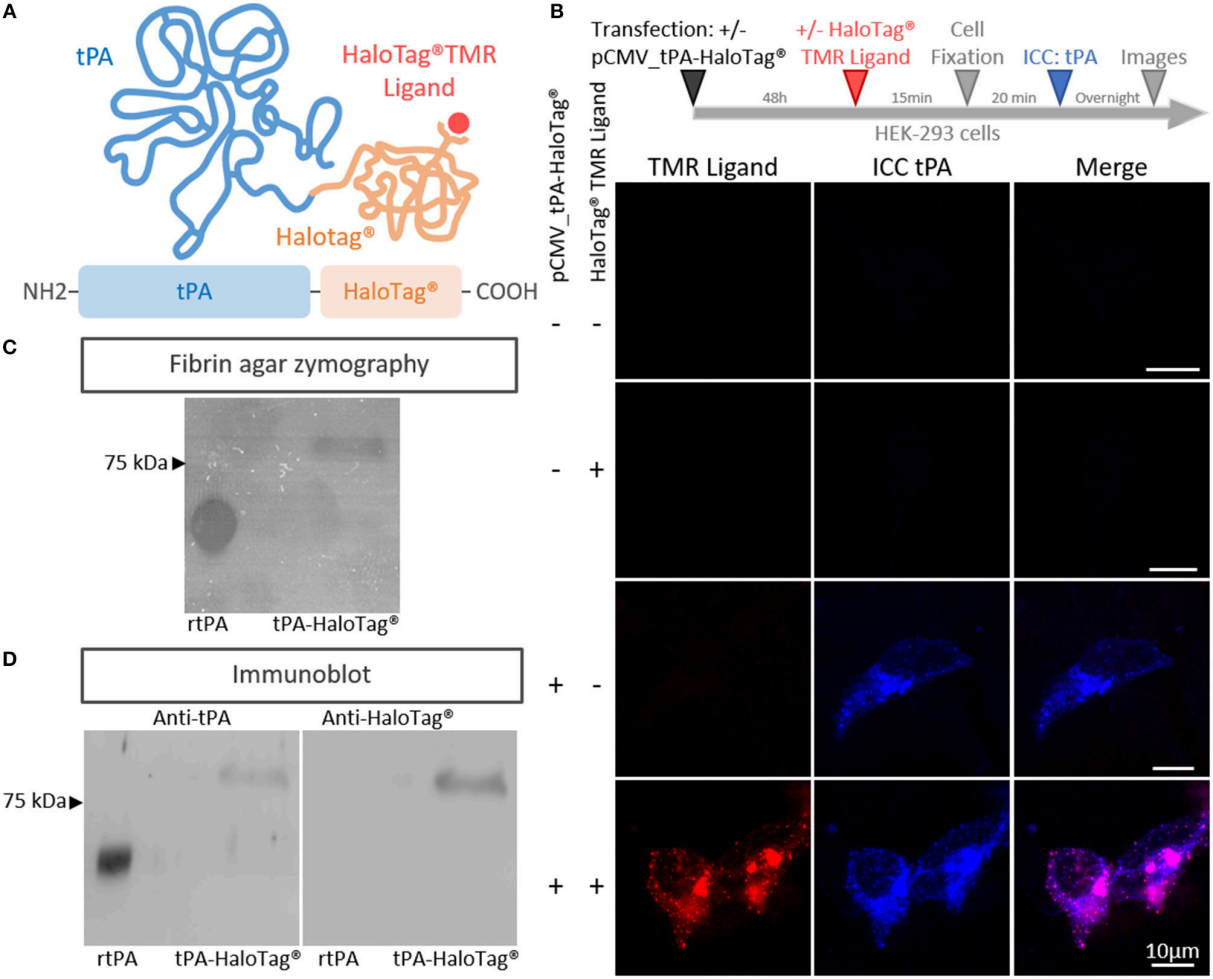
Generation of the tPA-HaloTag®. **(A)** Scheme of the tPA-HaloTag® protein: tPA in blue (69 kDa), HaloTag® in orange (33 kDa) and the specific fluorescent exogenous ligand (HaloTag® TMR Ligand) in red. **(B)** Representative confocal images of HEK-293 cells transfected (+) or not (–) with pCMV_tPA-HaloTag®, incubated 48 h later (+) or not (–) in the presence of the HaloTag® TMR ligand, followed by immunocytochemistry (ICC) raised against tPA (blue). Scale Bar: 10 μm. **(C)** Fibrin–agar zymography showing the fibrinolytic activity of recombinant tPA (rtPA) as control and tPA-HaloTag® produced by HEK 293 transfected cells. **(D)** Immunoblots of recombinant tPA (rtPA) and proteins from HEK-293 cells culture transfected with pCMV_tPA-HaloTag® revealed with antibodies raised against either tPA or HaloTag®. Control tPA (rtPA).

**Figure 2:**
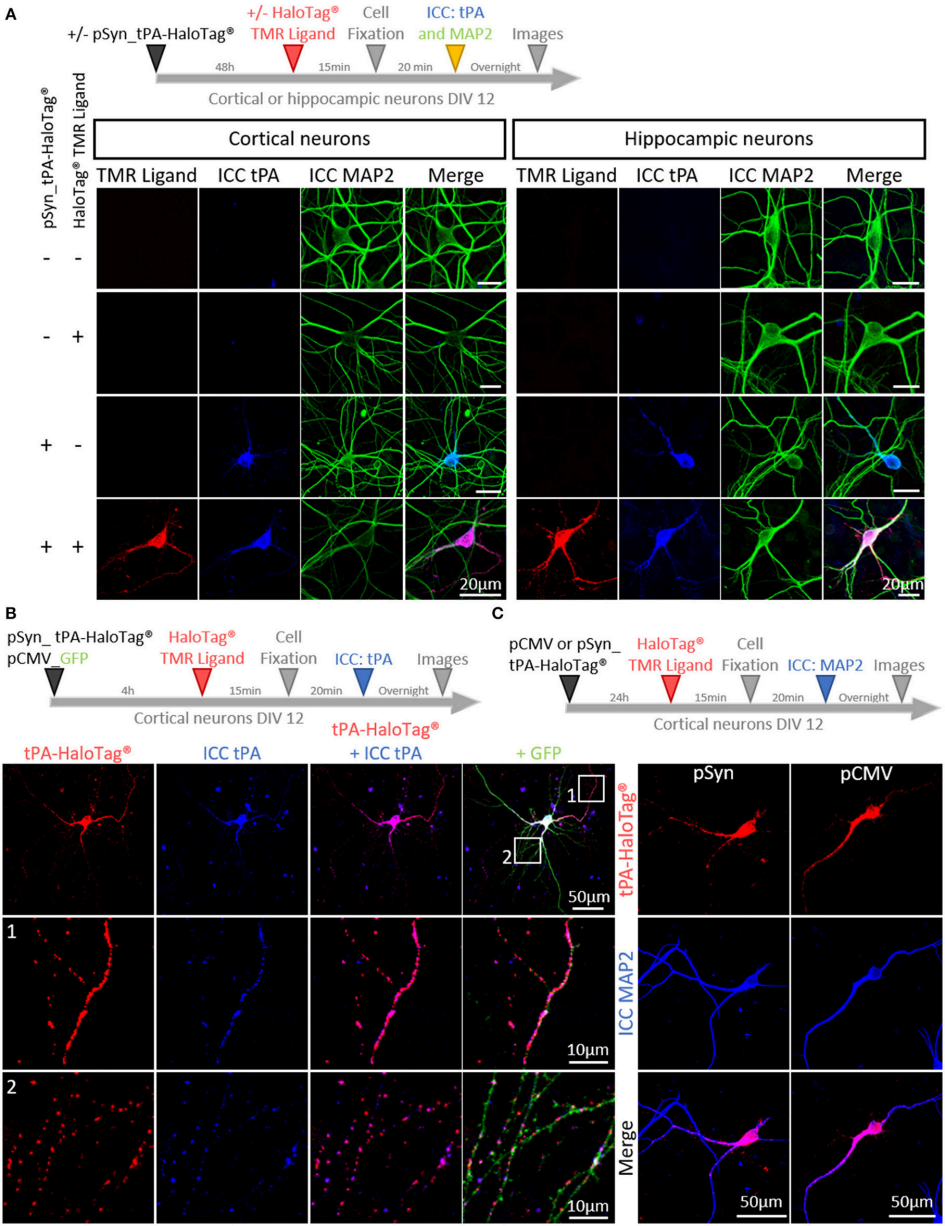
Characterization of tPA-HaloTag® plasmids. **(A)** Timeline of the experiments. Representative confocal images of cortical and hippocampal neurons transfected (+) or not (–) at DIV12 (Day *In Vitro*) with pSyn_tPA-HaloTag® and exposed 48 h later (+) or not (–) to the HaloTag® TMR ligand, together with an immunocytochemistries (ICC) against tPA (blue) and MAP2 (green). Scale Bar: 20 μm. **(B)** Transfection of cortical neurons at DIV 12 with pSyn_tPA-HaloTag® (red) and pCMV_GFP (green) plasmids, exposed 4 h later to the HaloTag® TMR ligand and ICC performed to reveal tPA (in blue). Z-stack and scale Bar: 50 μm (whole neuron) and 10 μm (1 and 2: whole neuron zoom). **(C)** Timeline of the experiments. Transfections of pCMV_tPA-HaloTag® or pSyn_tPA-HaloTag® (both revealed in red) and immunochemistry raised against MAP2 (in blue) show similar neuronal expressions of tPA-HaloTag® with the two constructs. Scale of tPA-HaloTag® with the two constructs. Scale Bar: 50 μm (whole neuron).

In order to track neuronal tPA in live cells, we inserted a HaloTag® at its C-terminal end (tPA-HaloTag®, Figure 1A). The tag HaloTag® is a modified haloalkane dehalogenase designed to covalently bind chloroalkane coupled with fluorophore (HaloTag® ligands) (Encell et al., 2012). This technology offers a wide range of ligands allowing a multitude of applications (localization, pulse chase, protein interactions) (England et al., 2015). In our study, we used a cell permeable exogenous fluorescent ligands, the HaloTag® TMR Ligand (555Ex/585Em). When tPA-HaloTag® was expressed in HEK-293 cells as control, it was detected by either the “HaloTag® TMR Ligand” or an antibody raised against tPA in the intracellular compartment (Figure 1B). Accordingly, a recombinant proteolytically active tPA-HaloTag® was detected from protein extracts of transfected HEK-293 cells (69 kDa for tPA plus 33 kDa for the HaloTag®) (Figure 1C) also revealed with antibodies raised against either tPA or HaloTag® (Figure 1D). Control experiments were also performed from cortical and hippocampal mouse neuronal cultures (Figures 2A,B), confirming the neuronal expression of tPA-HaloTag®, revealed either by the HaloTag® TMR Ligand or with an antibody raised against tPA. Expression of tPA-HaloTag® were driven either by a viral promoter pCMV (CytoMegaloVirus) or by the neuronal specific promoter synapsin (pSyn), with similar profiles of expression (Figure 2C) which suggest that the two promoters can be used in parallel. Similarly, the position of the HaloTag®, either N-terminal or C-terminal of tPA did not change its neuronal distribution (Supplementary 1). Furthermore, cortical neurons in culture are able to activate tPA promotor (Supplementary 2).

**Figure 3:**
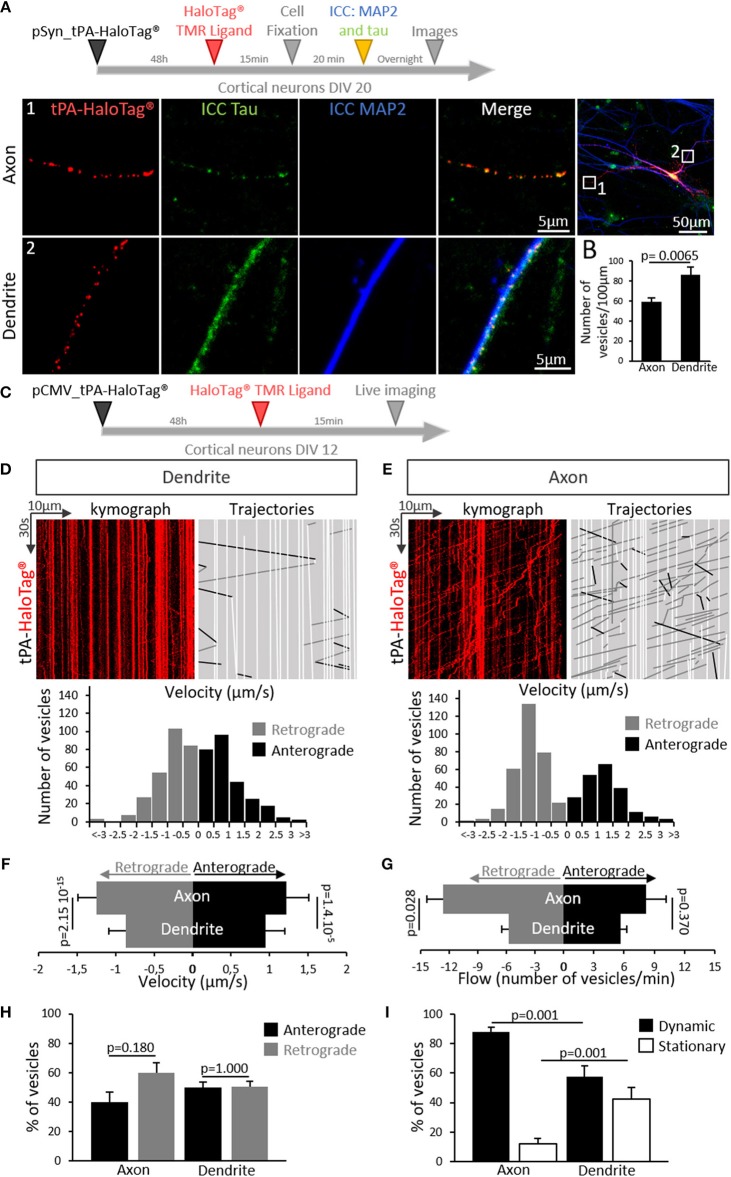
tPA is present and transported in axons and dendrites. **(A)** Timeline of the experiments. Representative z-stack confocal images of cortical neurons (at DIV 20) transfected with pSyn_tPA-HaloTag®, incubated in the presence of the HaloTag® TMR ligand (in red) and subjected to immunocytochemistries raised against tau (tubulin-associated unit, in green) and MAP2 (microtubule associated protein 2, in blue). tPA-HaloTag® is present in axons (1; MAP2 – and tau +) and in dendrites (2; MAP2 + and tau +). Scale Bar: 5 or 50 μm (whole neuron). **(B)** Quantification of the number of TMR positive puncta per 100 μm along the axons (59 puncta/100 μm) and dendrites (86 puncta/100 μm). *n* = 15 and *n* = 22 neurons respectively, from *N* = 6 independent cultures; *p* = 0.0065. **(C)** Timeline of experiments. **(D)** Transport of tPA-HaloTag® in cortical dendrites. Representative kymographs generated from dendrites (in red) and analyzed trajectories of vesicles are shown with a color code, black for anterograde, gray for retrograde and white for static vesicles. Down, representative diagrams of the distribution of vesicles velocities (μm/s) from seven analyzed kymographs (270 anterograde vesicles, 278 retrograde vesicles, *n* = 7 neurons from 5 independent cultures). **(E)** Transport of tPA-HaloTag® in cortical axons. Representative kymographs generated from axons and analyzed trajectories of vesicles are shown with the same color code viewed previously in **(D)**. Down, diagrams illustrate the distribution of vesicles velocities (μm/s) from five analyzed kymographs (209 anterograde vesicles, 317 retrograde vesicles from *n* = 5 neurons from 4 independent cultures). **(F)** Histograms recapitulate dendritic and axonal vesicles velocities (μm/s) in anterograde way (axons: 1.22 μm/s; dendrites: 0.95 μm/s) and in retrograde way (axons: 1.24 μm/s; dendrites: 0.87 μm/s). (Anterograde: 209 vesicles for axons and 270 for dendrites; *p* = 1.4.10^−5^. Retrograde 317 vesicles for axons and 278 for dendrites; *p* = 2.15.10^−15^, *n* = 5 and *n* = 7 neurons respectively, from *N* = 9 independent cultures). Student's *t*-test. **(G)** Histograms show the tPA-HaloTag® vesicles flow (number of vesicles per min) repartition in anterograde and retrograde directions in dendrites and axons on a time proportion (anterograde: 8 vesicles for axons and 5 for dendrites per min; *p* = 0.370. Retrograde 13 vesicles for axons and 6 for dendrites per min; *p* = 0.028. *N* = 5 and *N* = 7 neurons respectively, from *N* = 9 independent cultures). Mann-Whitney's *U test*. **(H)** Histograms showing the percentages of tPA positives vesicles repartition in both ways in axons (anterograde: 40%; retrograde: 60%, *p* = 0.180) and in dendrites (anterograde: 49%; retrograde: 51%, *p* = 1.0) (axons *N* = 5 and dendrites *N* = 7 neurons, from *N* = 9 independent cultures). Mann and Whitney' *U test*. **(I)** Histograms showing percentages of dynamic tPA positives (TMR positive puncta, in red) vesicles in axons (88%) and dendrites (57%); *p* = 0.001, and the percentages of stationary tPA vesicles in axons (12%) and in dendrites (43%); *p* = 0.001, (*N* = 10 neurons for axons and *N* = 7 neurons for dendrites, from *N* = 6 independent cultures). Student's *t*-test.

**Figure 4:**
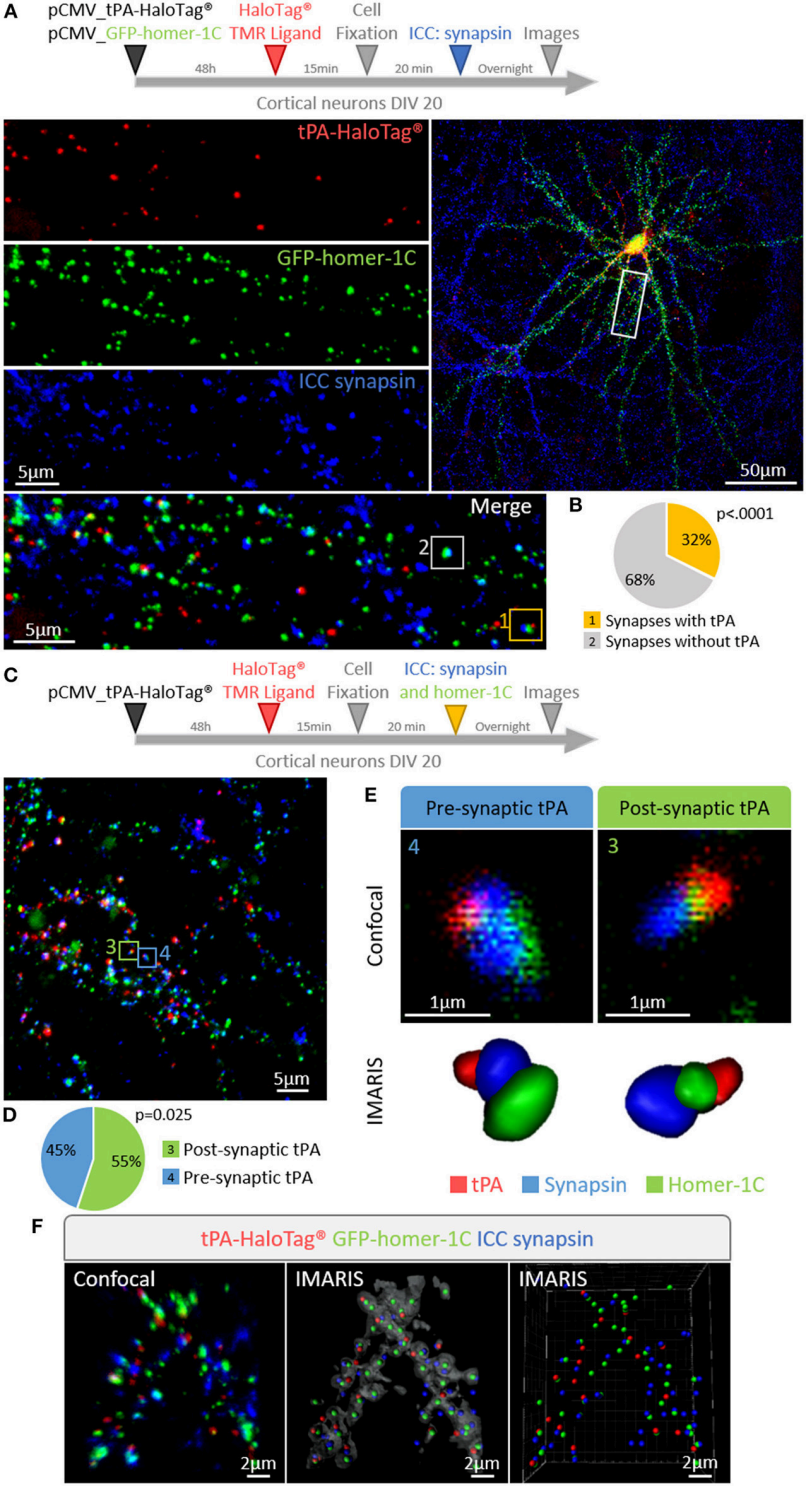
tPA is present in pre- and post-synaptic elements. **(A)** Timeline of the experiments. Representative z-stack confocal images of cortical neurons (at DIV 20) co-transfected with pCMV_tPA-HaloTag® and pCMV_GFP-homer-1C plasmids followed by immunocytochemistry raised against synapsin. tPA-HaloTag® (TMR ligand, in red) is present at synapses revealed by the presence of GFP-homer-1C (post-synaptic protein, green) and synapsin (pre-synaptic protein, blue). Scale Bar: 5 or 50 μm (whole neuron). **(B)** Representative diagrams showing the percentages of synapses containing or not tPA-HaloTag®. TMR staining (i.e., tPA) is present in 32% of all synapses (yellow frame: 1) and 68% of synapses are negative for tPA (white frame: 2), *N* = 21 neurons from *N* = 6 independent cultures and *p* < 0.0001. **(C)** Timeline of the experiments. Cortical neurons at DIV 20 were transfected with pCMV_tPA-HaloTag® plasmid, ICC raised against synaspin and homer-1C were performed. Representative z-stack confocal images and diagrams show the percentages of tPA located either pre or post-synaptically. Scale Bar: 5 μm**. (D)** 55% of all synaptic tPA is post-synaptic (green frame in confocal image; 3) and 45% of all synaptic tPA is pre-synaptic (blue frame in confocal image; 4). *N* = 10 neurons from *N* = 2 independent cultures and *p* = 0.025. **(E)** IMARIS reconstructions from confocal images C of pre-synaptic tPA (blue frame; 4) and post-synaptic tPA (green frame; 3). **(F)** Representative 3D-confocal images of co-transfected cortical neurons (at 3D-confocal images of co-transfected cortical neurons (at DIV20) with pCMV_tPA-HaloTag® (in red) and pCMV_GFP-homer-1-C (in green) plasmids followed by immunocytochemistry raised against synapsin (in blue). IMARIS reconstruction from confocal images. Scale Bar: 2 μm.

We then aimed to determine whether the expression of neuronal tPA was polarized. Primary cultures of mouse cortical neurons (DIV 20) were transiently transfected for 48 h with the pSyn_tPA-HaloTag® plasmid and subjected to immunostainings for either MAP2 (microtubule-associated protein 2) or tau (tubulin-associated unit) (Figure 3A). We found the presence of tPA in dendrites (MAP2 and tau positive) and axons (tau positive only), with a mean number of 86 puncta of tPA-HaloTag® per 100 μm in dendrites vs. 59 puncta per 100 μm in axons (*n* = 22 neurons and *n* = 15 neurons respectively, from 6 independent neuronal cultures, *p* = 0.0065, Mann and Whitney' *U test*). The distribution of neuronal tPA was thus not fully polarized, although the number of tPA dots was higher (+45%) in dendrites than in axons (Figure 3B). We then investigated the motility of tPA-containing vesicles in axons and dendrites (Figure 3C). Kymograph analyses of the trajectories showed that tPA-containing vesicles moved bidirectionally in axons and dendrites (Figures 3D,E). Interestingly, the velocity of vesicles, both retrograde and anterograde, was higher in axons than in dendrites; Anterograde: 1.21 μm/s in axons vs. 0.94 μm/s in dendrites; *p* = 1.4.10^−5^, Student's *t*-test, (*n* = 270 and 209 vesicles respectively, measured from 5 independent neurons of 4 independent neuronal cultures)–Retrograde: 1.24 μm/s in axons vs. 0.86 μm/s in dendrites *p* = 2.15.10^−15^, Student's *t*-test (*n* = 278 and 317 vesicles respectively, measured from 7 independent neurons of 5 independent neuronal cultures) (Figure 3F). The number of mobile vesicles was higher in axons (mainly retrograde) than in dendrites (anterograde: 8 vesicles/min in axons vs. 5 vesicles/min in dendrites; *p* = 0.370, Mann-Whitney's *U test* and retrograde: 13 vesicles/min in axons vs. 6 vesicles/min in dendrites; *p* = 0.028, Mann-Whitney's *U test, n* = 5 and 7 neurons respectively, from 9 independents neuronal cultures) (Figure 3G and Supplementary movies 1, 2). Interestingly, although all axonal tPA-containing vesicles were mobile, around half of the dendritic tPA-containing vesicles were stationary (mobile/stationary: for axons: 88/12%, for dendrites: 57/43%) (Figures 3H,I) (*n* = 10 neurons for axons and *n* = 7 neurons for dendrites from 6 independent neuronal cultures, *p* = 0.001, Student's *t*-test). To characterize further the location of tPA at the synapse, neurons were co-transfected with a vector encoding for the postsynaptic marker GFP-homer-1C and immunostained for the presynaptic marker synapsin (Figure 4A). We found that 32% of synapses were tPA-positives (*n* = 21 neurons from 6 independent neuronal cultures, *p* < 0.0001 Mann and Whitney' *U test*, Figure 4B). Furthermore, 55% of synaptic tPA was located post-synaptically, as shown in neurons immunostained for homer-1C and synapsin (Figures 4C,F, *n* = 10 neurons from 2 independent neuronal cultures, *p* = 0.025, Mann and Whitney' *U test*).

### tPA Is Contained in Vesicles Displaying a Constitutive Post-synaptic Release

**Figure 5:**
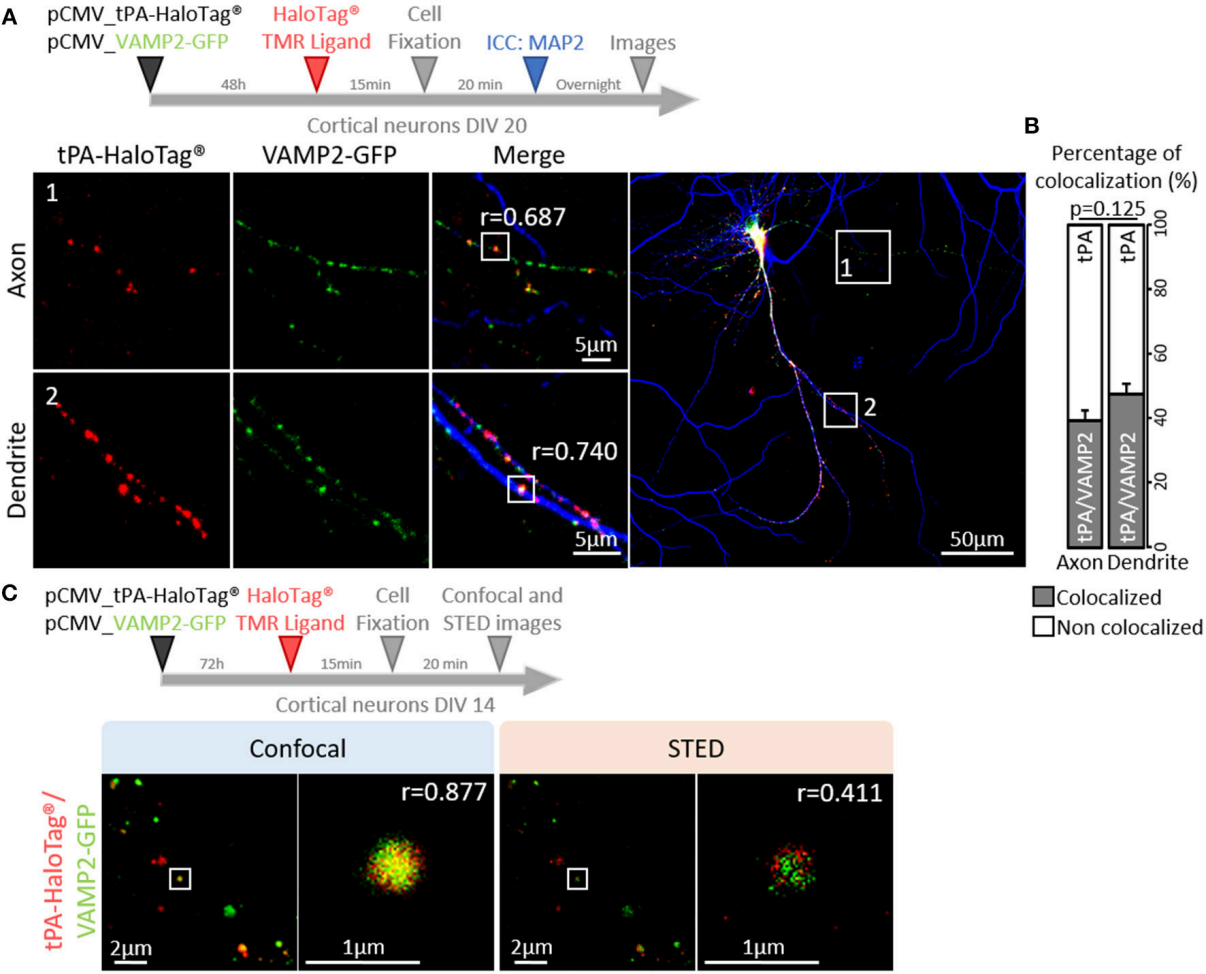
tPA is associated with VAMP2 proteins. **(A)** Timeline of the experiments. Representative z-stack images of co-transfected cortical neurons (at DIV20) with pCMV_tPA-HaloTag® (TMR ligand, in red) and pCMV VAMP2-GFP (in green), followed by ICC raised against MAP2 (in blue). tPA-HaloTag® colocalizes with VAMP2-GFP (exosome) in axons (MAP2-; frame 1) and in dendrites (MAP2+; frame 2) with Pearson's coefficients, respectively: *r* = 0.687 and *r* = 0.740. Scale Bar: 5 or 50μm (whole neuron). **(B)** Representative diagrams of the percentages of colocalization between tPA-HaloTag® and VAMP2-GFP in function of total tPA-HaloTag® puncta. There are 39% of colocalization in axons and 48% in dendrites (*p* = 0.125). **(C)** Representative images of co-transfected cortical neurons cortical neurons (at DIV14) with pCMV_tPA-HaloTag® (reveals by TMR ligand, in red) and pCMV_VAMP2-GFP (in green). tPA-HaloTag® and VAMP2-GFP colocalize, when revealed by confocal and super-resolution microscopy: Stimulated Emission Depletion (STED) with Pearson's coefficients, respectively: *r* = 0.877 and *r* = 0.411. Scale Bar: 2 or 1 μm.

**Figure 6:**
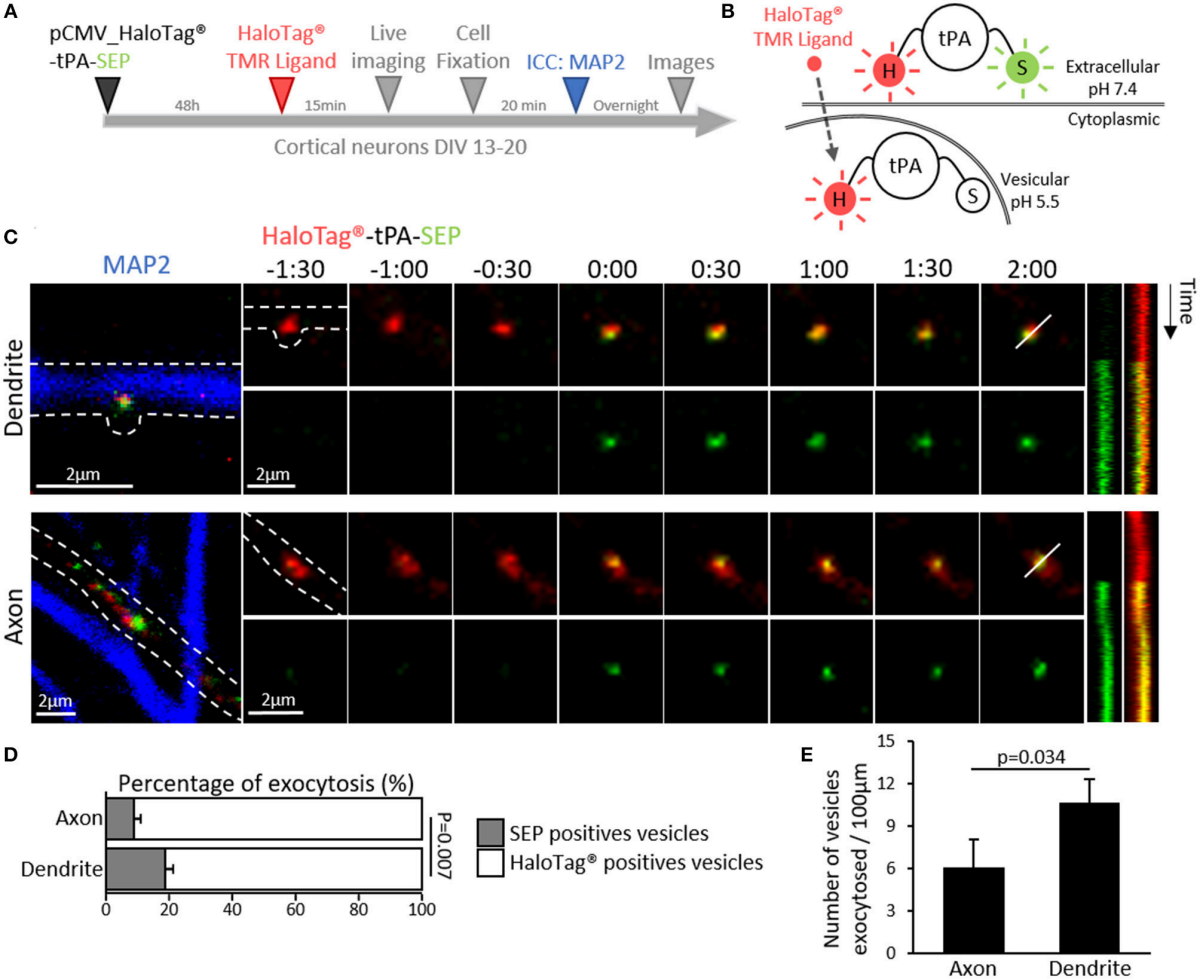
Dendritic and axonal release of tPA. **(A)** Timeline of the experiment. **(B)** Schematic diagrams of a dual color reporter for neuronal tPA. tPA fused to a HaloTag® site and the superecliptic pHluorin (SEP) allows simultaneous visualization of total (HaloTag®) and extraneuronal (SEP) tPA molecules. **(C)** Representative z-stack confocal images of transfected cortical neurons (DIV12) with pCMV_HaloTag®-tPA-SEP plasmid (TMR ligand; in red and SEP; in green), time-lapse were acquired in 2 dimensions every 30 s (*t* = 0 was assigned of exocytosis; Time is in min:sec). After live imaging ICCs were performed to reveal MAP2 (in blue) and thus to differentiate axons from dendrites (an example of each are shown). Respective kymographs of HaloTag®-tPA-SEP exocytosis are shown. Scale bar represents 2 μm. **(D)** Histograms show the percentages of exocytotic tPA-containing vesicles (SEP positives puncta, in green) compared to the total pool of tPA-containing vesicles (TMR positives puncta, in red) in axons (9%) and in dendrites (19%) (*N* = 16 and *N* = 14 neurons respectively, from *N* = 7 independent cultures, *p* = 0.007). **(E)** Schematic histograms of the number of vesicles exocytosed per 100μm in axons (6) and dendrites (11) (*N* = 13 and *N* = 11 neurons respectively, from *N* = 5 independent cultures, *p* = 0.034).

Trafficking of synaptic vesicles includes their formation, intracellular movement and exocytosis. Each of these processes is driven by dedicated proteins, named V-SNARE (Vesicular Soluble N-ethylmaleimide-sensitive-factor Attachment protein) (Chaineau et al., 2009). VAMP2 (aka Synaptobrevin 2) is the main v-SNARE of synaptic vesicles. Neurons (DIV 20) expressing tPA-HaloTag® were thus co-transfected with GFP-tagged VAMP2 (Figure 5A) and subjected to immunostainings against MAP-2. In axons, VAMP2-GFP positive spots co-localized with 39% of the total tPA puncta (Pearson's coefficient *r* = 0.687). In dendrites VAMP2-GFP positive spots co-localized with 48% of the total tPA puncta (*r* = 0.740) (Figures 5 A,B; *n* = 6 and *n* = 10 neurons respectively, from 6 independent neuronal cultures, *p* = 0.125, Mann and Whitney' *U test*). The presence of VAMP2 positive vesicles containing tPA was confirmed by using high resolution STED imaging (Figure 5C). We then engineered a dual color reporter HaloTag®-tPA-SEP construct with the pH sensitive GFP variant super ecliptic PHluorin (SEP) as a sensor of tPA-containing vesicles exocytosis (Figures 6A,B). Control treatment with ammonium chloride to buffer HaloTag®-tPA-SEP-containing vesicles led to the switch of GFP negative tPA-containing vesicles (normally acid) to GFP positive tPA-containing vesicles (NH_4_Cl inducing capture of protons), as previously reported (Shen et al., 2014) (Supplementary 3). Using live cell imaging (Figure 6C) we observed that, under resting conditions, the constitutive exocytosis of tPA was higher in dendrites than in axons (11 SEP positive vesicles per 100 μm in dendrites (19% of the total number of tPA positive vesicles) vs. 6 in axons (9% of the total number of tPA positive vesicles, *p* = 0.007, Mann and Whitney' *U test*), *n* = 11 and *n* = 13 neurons respectively, from 5 independent neuronal cultures, *p* = 0.034, Mann and Whitney' *U test*; Figures 6D,E). We also examined exocytosis after neuronal depolarization (KCl treatment; 50 mM) and we found an increase of the tPA release in both dendrites and axons (*p* = 0.025 and *p* = 0.026, respectively) with an increase of the percentage of stationary vesicles (Supplementary 4).

### tPA Is Contained in Rabs Positive Vesicles

**Figure 7:**
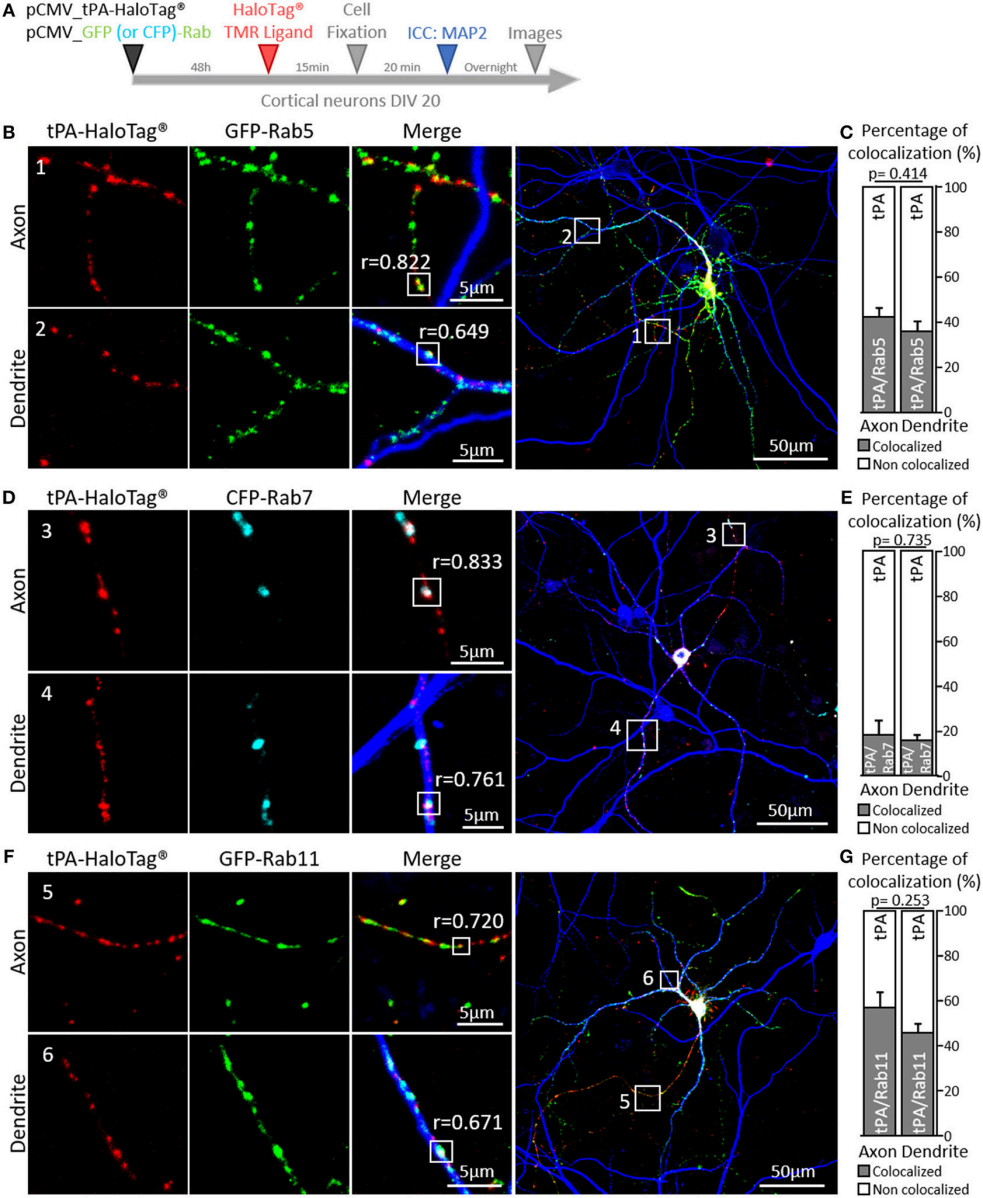
tPA is associated with Rabs proteins. **(A)** Timeline of experiments. **(B)** Representative z-stack images of co-transfected cortical neurons (DIV20) with pCMV_tPA-HaloTag® (TMR ligand, in red) and pCMV_GFP-Rab5 (in green), followed by ICCs raised against MAP2 (in blue). tPA-HaloTag® colocalizes with GFP-Rab5 (early endosome) in axons (MAP2–; frame 1) and in dendrites (MAP2+; frame 2) with Pearson's coefficients respectively, of *r* = 0.822 and *r* = 0.649. **(C)** Representative diagrams of the diagrams of the percentages of colocalization between tPA-HaloTag® and GFP-Rab5 in function of total tPA-HaloTag® puncta. There are 42% of colocalization in axons and 36% in dendrites (*N* = 3 and *N* = 8 neurons respectively, from 4 independent cultures, *p* = 0.414). **(D)** Representative images of co-transfected cortical neurons (at DIV20) with pCMV_tPA-HaloTag® (reveals by TMR ligand, in red) and pCMV_CFP-Rab7 (in cyan), followed by ICCs raised against MAP2 (in blue). tPA-HaloTag® colocalizes with CFP-Rab7 (late endosome and lysosome degradation) in axons (MAP2–; frame 3) and in dendrites (MAP2+; frame 4) with Pearson's coefficients respectively of *r* = 0.833 and *r* = 0.761). **(E)** Representative diagrams of the percentages of colocalization between tPA-HaloTag® and CFP-Rab7 in function of total tPA-HaloTag® puncta. There are 18% of colocalization in axons and 16% in dendrites (*N* = 3 and *N* = 10 neurons respectively, from 4 independent cultures and *p* = 0.735). **(F)** Representative z-stack images of co-transfected cortical neurons (at DIV 20) with pCMV_tPA-HaloTag® (TMR ligand, in red) and pCMV_GFP-Rab11 (in green), followed by ICCs raised against MAP2 (in blue). tPA-HaloTag® colocalizes with GFP-Rab11 (recycling endosome) in axons (MAP2–; frame 5) and in dendrites (MAP2+; frame 6) with Pearson's coefficients respectively, of *r* = 0.720 and *r* = 0.671. **(G)** Representative diagrams of the percentages of colocalization between Representative diagrams of the percentages of colocalization between tPA-HaloTag® and GFP-Rab11 in function of total tPA-HaloTag® puncta. There are 57% of colocalization in axons and 46% in dendrites (*N* = 6 and *N* = 7 neurons respectively, from 2 independent cultures and *p* = 0.253). For all images: scale Bar: 5 or 50 μm (whole neuron).

Additional experiments were performed by co-expressing GFP-Rab5 (early endosome), CFP-Rab7 (late endosome) or GFP-Rab11 (recycling endosome), which play complementary functions in recycling of synaptic molecules (Stenmark, 2009). We found colocalization of tPA-HaloTag® with: i/ GFP-Rab5 puncta in axons (42% of the total tPA puncta, *r* = 0.822) and dendrites (36% of the total tPA puncta, *r* = 0.649) (Figures 7B,C; *n* = 3 and *n* = 8 neurons respectively, from 4 independent neuronal cultures, *p* = 0.414, Mann and Whitney' *U test*), ii/ CFP-Rab7 in axons (18% of the total tPA puncta, *r* = 0.833) and dendrites (16% of the total tPA puncta, *r* = 0.761) (Figures 7D,E; *n* = 3 and *n* = 10 neurons respectively, from 4 independent neuronal cultures, *p* = 0.735, Mann and Whitney' *U test*) and iii/ GFP-Rab11 in axons (57% of the total tPA puncta, *r* = 0.720) and dendrites (44% of the total tPA puncta, *r* = 0.671) (Figures 7F,G; *n* = 6 and *n* = 7 neurons respectively, from 2 independent neuronal cultures, *p* = 0.253, Mann and Whitney' *U test*).

## Discussion

tPA was first shown to be produced and released in the circulation by endothelial cells, where it plays roles in inflammation and fibrinolysis (Hoylaerts et al., 1982; Zhang et al., 1999). In addition to its roles in the vascular system, tPA plays crucial functions in the central nervous system (CNS) acting either as an enzyme or as a growth-factor-like molecule (Thiebaut et al., 2018). Thus, tPA is involved in several physiological and pathological CNS processes, including corticogenesis, neuronal survival, learning and memory, anxiety, epilepsy, stroke and Alzheimer's disease (Qian et al., 1993; Baranes et al., 1998; Madani et al., 1999; Pawlak et al., 2003; Alvarez et al., 2013; Oh et al., 2014; Hébert et al., 2017; Pasquet et al., 2018). Its neuronal functions are achieved through plasminogen-dependent or plasminogen-independent effects (Melchor and Strickland, 2005).

Neurons represent an ultimate example of polarized cells with a complex network of membrane trafficking pathways which is necessary to allow efficient communication over long distances (Goldstein and Yang, 2000). Axonal and dendritic transports are essentials for neuronal survival and maturation. One of the most convincing demonstration of the crucial role of axonal transport in neuronal homeostasis is the causal link between defects in this process and neurodegeneration (Holzbaur, 2004). Cortical and hippocampal neurons can express endogenous tPA *in vivo* and *in vitro* (Fernández-Monreal et al., 2004; Louessard et al., 2016), in this study, we also demonstrate that neurons can activate the tPA promoter *in vitro*. Moreover, we have previously shown that tPA is mainly expressed in excitatory neurons and more particularly present in a subset of pyramidal glutamatergic neurons (Louessard et al., 2016). tPA has been identified in the presynaptic terminals within dense-core vesicles (Lochner et al., 2006). A previous study also indicated that tPA could activate the mechanism of endocytosis of synaptic vesicles (Wu et al., 2015).

In the present study, by studying the intra neuronal dynamics of tPA in cortical neurons, we showed that tPA is localized in closed proximity with VAMP2 positive vesicles in axons and dendrites. VAMP2 is known to mediate the exocytosis of synaptic vesicles in neurons and neuroendocrine cells (Schiavo et al., 1992). The use of confocal imaging is a possible limitation of this study, allowing us to reveal a synaptic localization of tPA, without affirming a vesicular colocalization. Although highly dynamic in axons (1.22 μm/s against 0.9 μm/s in dendrites), tPA-containing vesicles are more static in dendrites, possibly stopped at the post-synapse prior to their release. These data are in agreement with the literature for the velocity of dense-core vesicles (Lochner et al., 1998; Kwinter et al., 2009). After neuronal stimulation (KCl or NMDA exposures), there is an increase of the exocytosis of tPA in cultured cortical neurons (Nicole et al., 2001; Fernández-Monreal et al., 2004). Although the spontaneous release of tPA is more important in dendrites than in axons (Figure 6E), KCl treatment mainly promotes its axonal release (Supplementary 4E,G). This finding is consistent with the fact that for their maturation neurons need to release proteases such as tPA, plasmin or metalloproteinases to degrade the extracellular matrix, a process needed for spines formation (Seeds et al., 1992; Kim et al., 2017). This spontaneous release of tPA could be also involved in the maturation of pro-BDNF into mBDNF (via plasmin formation) (Pang et al., 2004; Briens et al., 2017) or in the interaction of tPA with various receptors at the synapse such as NMDAR (Lopez-Atalaya et al., 2008), LRP-1 (An et al., 2008), Annexin-II (Hajjar et al., 1994) to mediate its neuronal functions (Chevilley et al., 2015), including late phase of Long-Term Potentiation (LTP) (Qian et al., 1993; Baranes et al., 1998; Pang et al., 2004). A axonal release of tPA-containing vesicles was also observed, but at a lower level. Endocytosis is an important cellular mechanism, it is responsible of recycling and degradation of molecules (Sudhof, 2004; Stenmark, 2009). Here we show that the tPA can be stored in early endosome (Rab5 positive vesicles), late endosome (Rab7 positive vesicles) and recycling endosome (Rab11 positive vesicles). Several studies have shown that tPA can be recaptured by clathrin dependent endocytosis *via* LRP receptors (Narita et al., 1995; Benchenane et al., 2005; Briens et al., 2017). It is then interesting to note that such distribution displays similarities with the trafficking of the neuronal BDNF (Hartmann et al., 2001; Brigadski et al., 2005; Bucci et al., 2014; Shimojo et al., 2015). One hypothesis could be a non-specific internalization of tPA by macropinocytosis, as previously reported for BDNF (Philippidou et al., 2011). This type of endocytosis is important for neuronal survival thank to a retrograde transport of these proteins to the nucleus (Kononenko et al., 2017). These data are in agreement with the present retrograde transport of tPA in axons show in this study.

Since decades, we know that neuronal proteins storage is concentrated in dense core vesicles (DCV). It is also admitted that the neuronal tPA circulates within these vesicles as others proteins implicated in neuronal plasticity and neuronal transmission, such as BDNF and neuropeptides (Kwinter et al., 2009). All of them play a role, directly or indirectly in synaptic functions. Our present data confirm these observations, with tPA possibly contained in VAMP2 positive vesicles with a diameter similar to this of DCV. Our data show an opening of fusion pore, without clearly demonstrating a release of tPA, which could be explained by a kiss-and-run phenomenon (He et al., 2006). Postsynaptic DCV exocytosis is clearly define to be driven by Ca^2+^/Calmodulin dependent protein kinases (CaMK) (Shakiryanova et al., 2007; Wu et al., 2017; Stucchi et al., 2018). Here we revealed DCV tPA positives vesicles with their constitutive exocytosis higher in dendrites than in axons. These findings agree with the literature concerning exocytosis of DCV (de Wit et al., 2009; van de Bospoort et al., 2012). This dendritic release of tPA could be involved in the late phase of LTP *via* its interaction with the GluN1 subunit of synaptic NMDAR (Baranes et al., 1998; Lopez-Atalaya et al., 2008; Samson et al., 2008; Bouvier et al., 2018). In addition, tPA has a potential action on postsynaptic removal of AMPAR by an indirect phosphorylation of GluR1 subunit via a Ca^2+^/calmodulin-dependent protein kinase IIα (pCaMKIIα) dependent mechanism in a low Ca^2+^ level environment (Jeanneret et al., 2016). It could be also involved in the extracellular matrix degradation to promote spines formation (Seeds et al., 1992; Kim et al., 2017). Finally, tPA is also described as an indirect activator of pro-BDNF by a plasmin dependent mechanism involving astrocytes (Pang et al., 2004; Briens et al., 2017).

Altogether, we provide here a set of important information about the neuronal trafficking of tPA which should help to further understand brain functions of tPA.

## Ethics Statement

This study was carried out in accordance with the recommendations of EU guidelines (directive 2010/63/EU) and the French National Committee (2010/63) for care and use of laboratory animals.

The protocol was approved by the French National Committee (2010/63).

## Author Contributions

YH and DV have designed the study. SL, AV, LL, TG, and YH have performed the experiments. SL and AV have analyzed the data. SL, AV, YH, and DV wrote the manuscript. All authors have read and have approved the final manuscript.

### Conflict of Interest Statement

The authors declare that the research was conducted in the absence of any commercial or financial relationships that could be construed as a potential conflict of interest.
