# Supplementary material for: Post-synaptic Release of the Neuronal Tissue-Type Plasminogen Activator (tPA)
Source: Front Cell Neurosci. 2019 Apr 24;13:164. doi: 10.3389/fncel.2019.00164 (PMC6491899; doi:10.3389/fncel.2019.00164)
Supplement: Supplementary file 3 [file Data_Sheet_1.PDF]

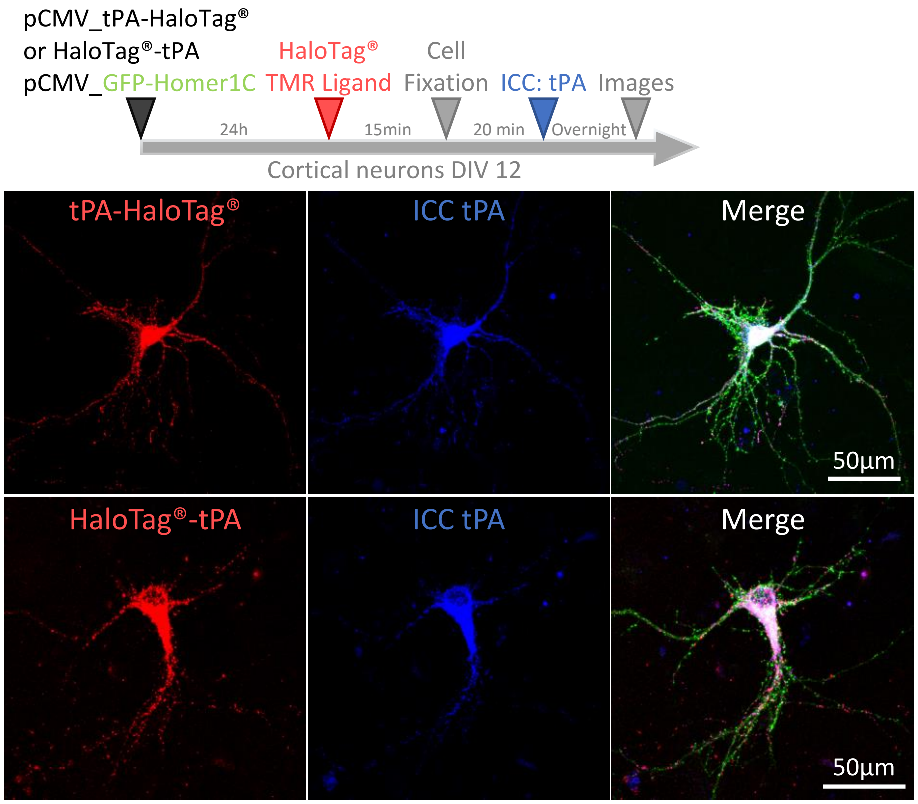

Supplementary 1: Characterization of tPA-HaloTag® plasmid. Timeline of the experiments. Co-Transfections of pCMV_tPA-HaloTag® or pCMV_HaloTag®-tPA (both in red) with pCMV_GFP-Homer-1C (in green) and immunochemistry raised against tPA (in blue) show that the position of the HaloTag®, either N-terminal or C-terminal of tPA did not change its neuronal distribution. Scale Bar: 50µm (whole neuron).

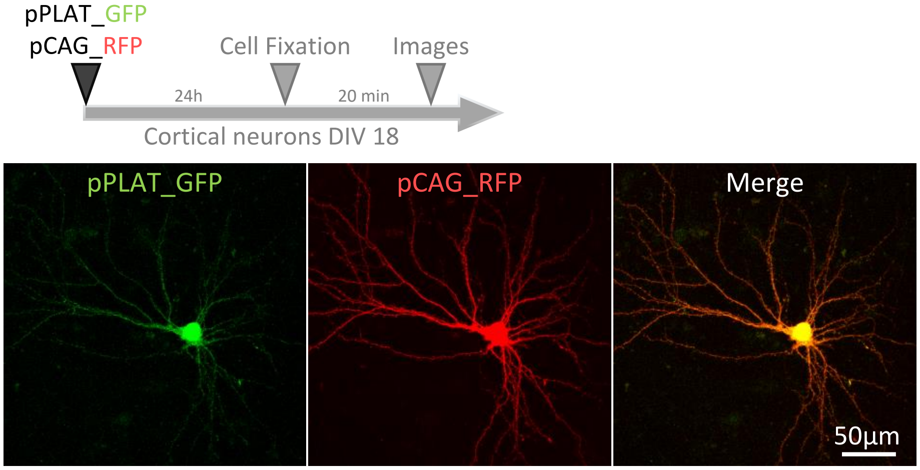

Supplementary 2: Endogenous expression of tPA in neurons. Timeline of the experiments. Representative z-stack confocal images of co-transfected cortical neurons (at DIV18) with pPLAT_GFP (tPA promotor, in green) and pCAG_RFP (control of transfection, in red). Cortical neurons can express GFP by activation of the tPA promotor. Scale Bar: 50 µm (whole neuron).

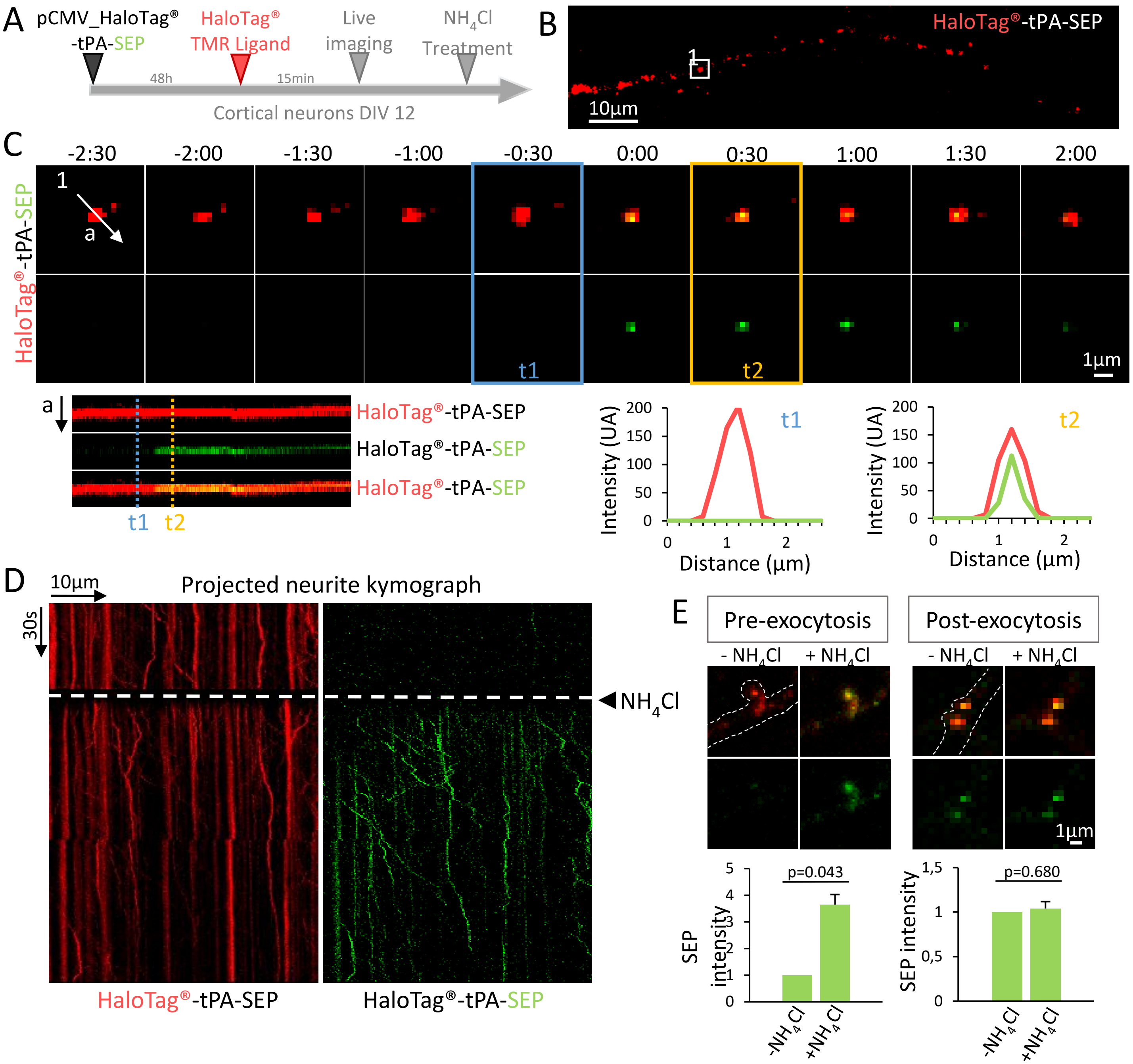

Supplementary 3: HaloTag®-tPA-SEP functionality. (A) Timeline of the experiments. **(B)** Representative confocal z-stack of cortical neurite expressing HaloTag®-tPA-SEP. Scale bar: 10µm. **(C)** Timeline imaging of a HaloTag®-tPA-SEP vesicle (white frame in B) exocytosis in basal condition. SEP fluorescence variation is shown by timeline generated kymographs and fluorescence intensity graphs analysis at t1 and t2 stages (Time in min:sec and Scale bar: 1µm). **(D)** Projected neurite kymograph transfected with HaloTag®-tPA-SEP before, before and after NH_4Cl (50mM) treatment which reveals total intracellular SEP positives vesicles pool. **(E)** SEP fluorescence analysis before (pre-exocytosis) and after (post-exocytosis) exocytosis with or without NH_4Cl (50mM). (Pre-exocytosis (p=0.043) and post-exocytosis (p=0.680) n=5 neurons from N=4 cultures). Scale bar: 1 µm. Wilcoxon's test.

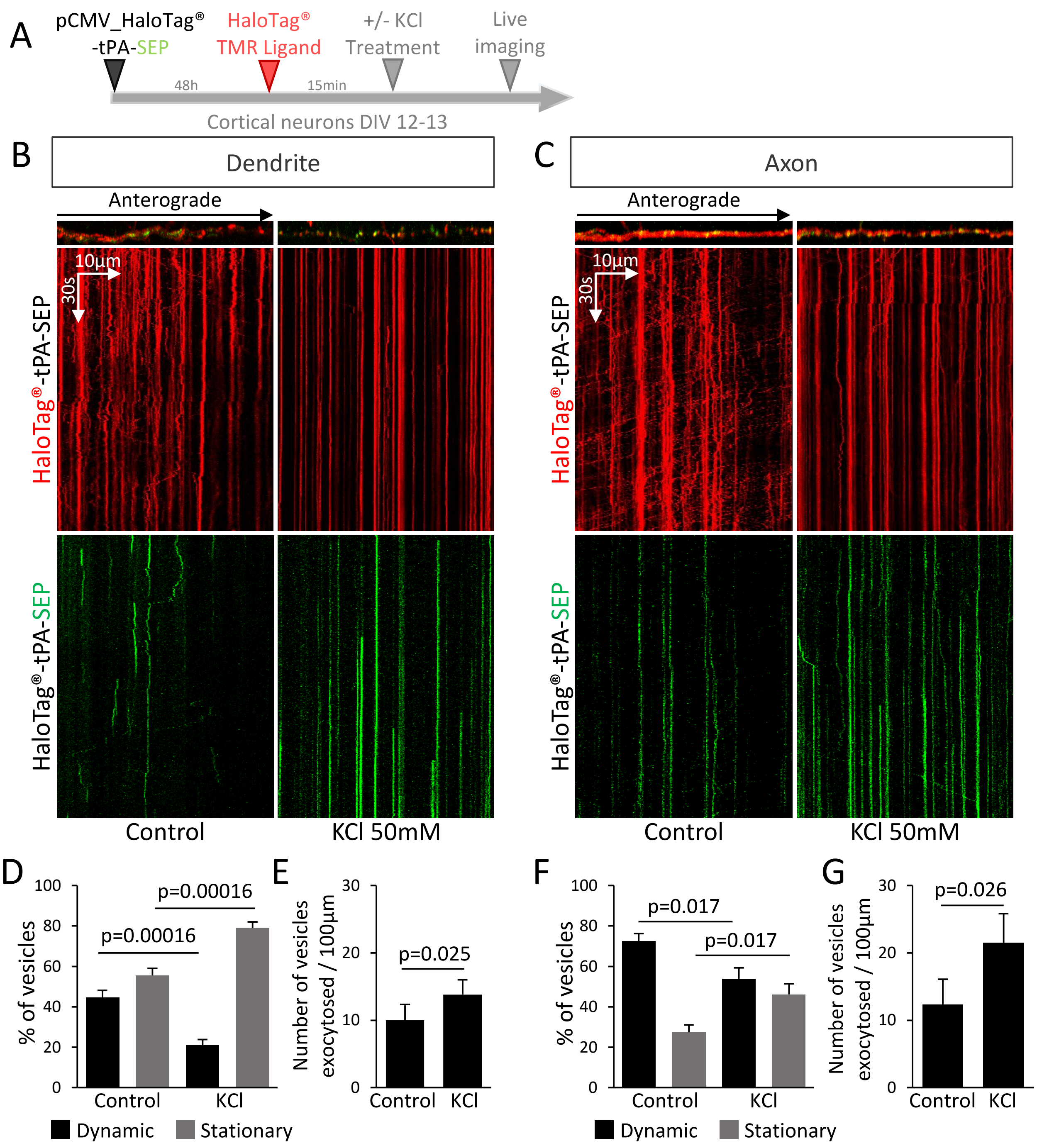

Supplementary 4: Exocytosis of tPA in response to KCl-induced neuronal activity. **(A)** Timeline of the experiments. **(B-C)** Representative kymographs of exocytosis with and without KCl treatment (50mM) on dendrites and axons of cultured cortical neurons (DIV12-13). Scale bar 10 µm, during 10 min. **(D)** Histograms showing the percentages of dynamic tPA positive vesicles in dendrites (TMR positive puncta, in red) in control (45%) and KCl treated cells (21%) and the percentages of stationary tPA positive vesicles in control (56%) and KCl treated cells (79%) (p=0.00016). **(E)** Schematic histograms of the number of vesicles exocytosed (SEP positives puncta, in green) per 100 µm in dendrites for control experiments (10) and after KCl treatments

(14) (p=0.025). **(D-E)** n= 23 neurons for control and n=10 neurons for KCl treatments, from N=3 and N=2 independent cultures, respectively. **(F)** Histograms showing the percentages of dynamic tPA positive vesicles in axons (TMR positive puncta, in red) in control (73%) and in KCl treated cells (54%) and the percentages of stationary tPA positive vesicles in control (27%) and in KCl treated cells (46%) (p=0.017). **(G)** Schematic histograms of the number of vesicles exocytosed (SEP positives puncta, in green) per 100 µm in axon for control experiments (12) and after KCl treatments (22) (p=0.026). **(F-G)** n= 10 neurons for control and n=10 neurons for KCl treatments, from N=3 and N=2 independent cultures, respectively. After KCl application we can observe an increase of stationary vesicles correlated to the number of vesicles exocytosed.

Supplementary movie 1: Trafficking of tPA positives vesicles in dendrites. Representative time lapse of dendritic trafficking (related to Figure 3) of cortical neurons (DIV13) transfected with pCMV_HaloTag®-tPA-SEP (TMR ligand, in red). Time lapse duration: 5min, scale bar: 10 µm and the white arrow points soma.

Supplementary movie 2: Trafficking of tPA positives vesicles in axon. Representative time lapse of axonal trafficking (related to Figure 3) of cortical neurons (DIV13) transfected with pCMV_HaloTag®-tPA-SEP (TMR ligand, in red). Time lapse duration: 5min, scale bar: 10 µm and the white arrow points soma.
